# Editorial: Mechanistic insights into plant biomechanical and biochemical adaptation to climate change

**DOI:** 10.3389/fpls.2025.1770085

**Published:** 2026-01-29

**Authors:** Marisabel Mecca, Erna Karalija, Bozeng Tang, Luigi Todaro, Nataša Lukić, Philip Linthilac, Julia J. Reimer

**Affiliations:** 1 Laboratory of Preclinical and Translational Research, Oncological Center of Basilicata (IRCCS-CROB), Rionero in Vulture, Italy; 2 Faculty of Science, University of Sarajevo, Sarajevo, Bosnia and Herzegovina; 3 School of Biological Sciences, Nanyang Technological University, Singapore, Singapore; 4 Department of Agricultural, Forestry, Food and Environmental Sciences, University of Basilicata, Potenza, Italy; 5 Institute of Molecular Genetics and Genetic Engineering, University of Belgrade, Belgrade, Serbia; 6 Department of Plant Biology, The University of Vermont, Burlington, VT, United States; 7 Faculty of Technology, Molecular Biosciences, University of Applied Sciences Emden/Leer, Emden, Germany

**Keywords:** crop plants, seed release, simulation models, sporangium, stability and spatial precision

The subject of plant biomechanics has traditionally been reserved for secondary tissues and woody stems, where tension and compression have macroscopic ramifications in reinforcing structural stability and overall strength, but many recent studies are showing that the development of primary tissues and meristems may also be subject to basic biomechanical principles that reflect the importance of physical messaging systems in plant development. In fact, it is becoming increasingly apparent that the developmental logic of the land plants, taken as a whole, may reflect a fundamental responsiveness to biomechanical signals that plays out in all stages of plant morphogenesis including the network of signaling systems that regulate sexual reproduction and gametogenesis. In this collection we bring together a number of different perspectives on plant biomechanics, illustrating the broad range of developmental behaviors that are driven by basic physical and mechanical principles and the evolution of physical responses to environmental pressures.

Climate change, through the combination of different stressors imposed on plants, is reshaping them at every level of biological organization. Rising temperatures, increased climatic variability, intensifying drought and heat episodes, and altered light and UV regimes all challenge the developmental, structural, and physiological limits of terrestrial plants (Zafar et al., 2024).

Understanding how plants sense, withstand, and adapt to these pressures requires an integrated perspective that spans molecular, biomechanical, and whole-plant physiological signalling pathways, as well as species-level ecological responses. This Research Topic, Mechanistic insights into plant biomechanical and biochemical adaptation to climate change, brings together studies that highlight these multiple scales of adaptation. A unifying theme across the contributions is that plant responses to stress cannot be understood through biochemical pathways alone. Since plant cells remain physically connected and mechanically coupled after division, development is inherently shaped by architectural, mechanical, and environmental constraints.

Lintilhac (2022) makes the point that our current understanding of plant development is built around molecular signaling pathways, underestimating the many positive attributes of biomechanical signaling. In the land plants, biomechanical signaling has many advantages over the stochastic and inherently labile nature of intracellular molecular signaling Putting this in the context of “Adaptation to climate change” Lintilhac points out that biomechanical signaling networks are likely to be more stable in the face of increased environmental instability.

Lintilhac (2024) interprets mechanistic aspects of reproductive differentiation as an evolutionary response to the environmentally exposed life-histories of the land plants, specifically focusing on the sporangium as an example of a stress-mechanical focusing device whose structure and growth create an isotropic singularity that is the original signal for the initiation of reproductive differentiation. Again, this kind of stress-mechanical signaling system is insensitive to environmental temperature fluctuation, and implies that land plants have evolved greater tolerance to environmental instability in a world whose environment is changing dramatically, and even beyond as Maeng and colleagues showed recently for mosses surviving of on space flights (Maeng et al., 2025).

In their study on *Abies concolor*, Wiczołek et al. provide new insights into the biomechanics of hygroscopic cone‐scale movements that regulate seed release in firs. Unlike pines, where motion is driven by a discrete bilayer hinge, *A. concolor* scales undergo broad, lamina-wide deformation. Overall, the study shows that hygroscopic movement in fir cones is driven by gradual changes across the scale lamina rather than by a single hinge region. This graded structure allows the scale to bend in a stable and adjustable manner while also providing useful ideas for designing humidity-responsive materials and improving our understanding of how conifers release their seeds.

As mentioned above, climate change is a multi-factorial process that influences terrestrial plants in different ways. Wang et al. model the projected distribution of *Oryza sativa*, one of the most important cereal crops. Using the R package Biomod2 they incorporate variables like min, max and mean temperature, and precipitation, to predict the rice distribution for China in 2050, 2070 and 2090 under various scenarios with or without changed UV radiation. They highlight the effects on agriculture and productivity. These investigations of spatial distribution of species in developing countries will become more significant (López-Tirado and Gonzalez-Andújar, 2023).

Adaptation by grafting is an ancient agricultural practice involving the transfer of metabolites in addition to transcripts, regulatory RNA and many more (Feng et al., 2024) from rootstock to scion and vice versa. Biermann et al. investigated heat stress tolerance in reciprocally grafted tomato (susceptible versus tolerant), highlighting the potential of using early developmental stages for screening. In addition, they combined phenotypic data (like yield or dry weight) with transcriptomic data indicating that the agronomically relevant trait(s) in tomato are controlled by an underlying signaling network complex, involving photosynthesis, heat shock proteins and ROS detoxification processes, as well as the modulation of energy-consuming developmental processes.

The published studies collected together in this research topic highlight different aspects of adaptation to climate change, from a broad, theoretical point of view and simulation to detailed insights in hygroscopic cone‐scale movements and adaptation by grafting. In summary, these articles show that land plant evolution has followed a structural logic that benefits the environmentally exposed life habits of the land plants.
